# Role of vitamins A, C, D, E in cancer prevention and therapy: therapeutic potentials and mechanisms of action

**DOI:** 10.3389/fnut.2023.1281879

**Published:** 2024-01-11

**Authors:** Wamidh H. Talib, Dima Abdulraheem Ahmed Jum’AH, Zeena Shamil Attallah, Mohanned Sami Jallad, Lina T. Al Kury, Rawan Wamidh Hadi, Asma Ismail Mahmod

**Affiliations:** ^1^Faculty of Allied Medical Sciences, Applied Science Private University, Amman, Jordan; ^2^Department of Clinical Pharmacy and Therapeutics, Applied Science Private University, Amman, Jordan; ^3^Department of Health Sciences, College of Natural and Health Sciences, Zayed University, Abu Dhabi, United Arab Emirates

**Keywords:** vit-E, vit-A, vit-D, combination anticancer therapy, vit-C

## Abstract

Cancer, a leading global cause of mortality, arises from intricate interactions between genetic and environmental factors, fueling uncontrolled cell growth. Amidst existing treatment limitations, vitamins have emerged as promising candidates for cancer prevention and treatment. This review focuses on Vitamins A, C, E, and D because of their protective activity against various types of cancer. They are essential as human metabolic coenzymes. Through a critical exploration of preclinical and clinical studies via PubMed and Google Scholar, the impact of these vitamins on cancer therapy was analyzed, unraveling their complicated mechanisms of action. Interestingly, vitamins impact immune function, antioxidant defense, inflammation, and epigenetic regulation, potentially enhancing outcomes by influencing cell behavior and countering stress and DNA damage. Encouraging clinical trial results have been observed; however, further well-controlled studies are imperative to validate their effectiveness, determine optimal dosages, and formulate comprehensive cancer prevention and treatment strategies. Personalized supplementation strategies, informed by medical expertise, are pivotal for optimal outcomes in both clinical and preclinical contexts. Nevertheless, conclusive evidence regarding the efficacy of vitamins in cancer prevention and treatment is still pending, urging further research and exploration in this compelling area of study.

## 1 Introduction

**Figure 1:**
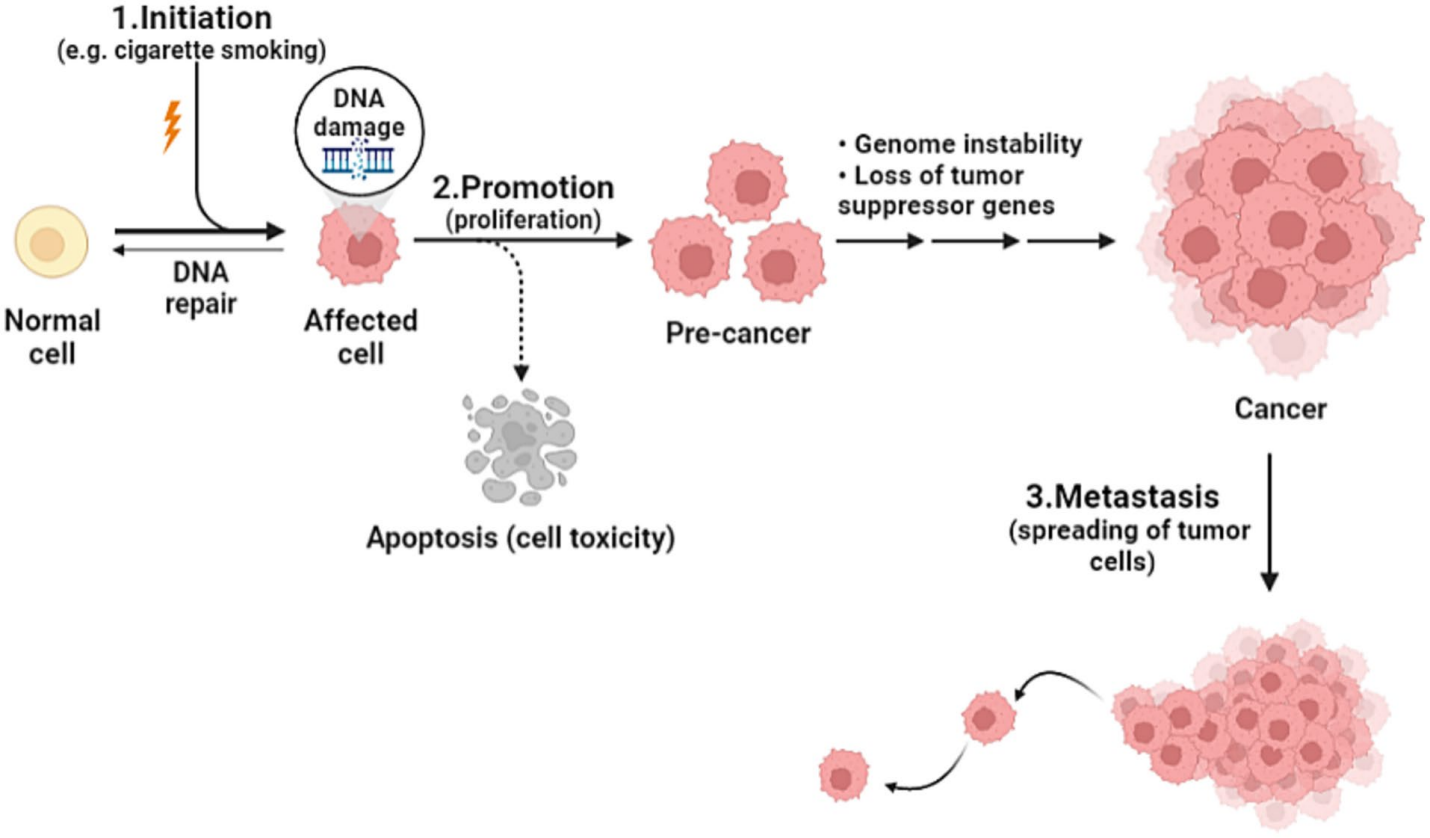
Cancer development steps.

Cancer is the health problem of the century, being the second leading cause of death after heart disease worldwide. As the world population is increasing, and as humanity have made such huge progress against causes of death that once killed people early in life, the number of cancer deaths has increased from around 5.7 million in 1990 to 8.8 million in 2017. It is true, the number of cancer deaths is increasing, but yet, individual death rates are falling (1). Cancer is defined as an unregulated proliferation and spread of cells in the body and can reach trillions in number. In a normal setting, human cells grow and multiply via cell division to form new needed cells. When this order breaks down, abnormal cells start to grow and multiply to develop abnormal tissues (2). The complicated prosperous nature of cancer is affected by various elements including genetic and environmental factors such as tobacco smoking, urbanization, and changing diet patterns. The genetic changes that lead to cancer can happen because of several reasons including errors that occur at cell division, DNA damage that may have been caused by substances in the environment, or genetic predispositions that were inherited from the parents. Each cancer is a unique combination of these genetic changes, and as cancer continues to develop, additional changes will occur (3). Studies devoted to establishing measures that focus on environmental factors have been implemented; nevertheless, little progress in favor of reducing cancer incidence has been made (4). In the domain of cancer biology, cancerous cells develop from healthy cells through a complex process known as malignant transformation, involving several key steps. Firstly, it begins by altering the genetic material of the cells, preparing it to become cancerous. The second step, promotion, is induced by agents known as promoters, which can include substances in the environment or certain medications. Promoters allow cells that were primed to become cancerous, and they do not affect cells that have not undergone initiation. The immune system is usually able to recognize cancer cells and destroy them before they duplicate, this explains why certain cancers are more likely to progress in people whose immune system is impaired. The third step is the spreading of cancer cells through the bloodstream or lymphatic system to nearby or distant locations; it can spread and invade surrounding tissues or organs (Figure 1). As it grows, nutrients are provided by their direct diffusion to cells, this further leads to increased cancer volume, destroying adjacent tissues (5). Understanding cancer’s cellular kinetics gives important insight when it comes to designing dosing schedules and timing intervals of treatment (6). Prevailed anticancer treatments such as chemotherapy, surgery, and radiotherapy have been challenging in terms of their adverse effects and disease progression, hence the humongous number of efforts to establish an alternative treatment regimen (7). The new approach is associated with vitamins and recently their anticipated anticancer effects have been considerably analyzed. Vitamins are essential nutrients for human metabolism, taking part in an essential function as coenzymes or enzymes in many imperative procedures for the regular functioning of the body, and it has been obvious that nutritional vitamins essentially contribute to human disease and healing (7). In a comprehensive review of 65 clinical trials involving cancer patients, the safety of various dietary supplements, particularly vitamins, was assessed. The findings revealed that vitamins, among other supplements, were generally safe for cancer patients, emphasizing the importance of continued research on the impact of vitamins in cancer treatment (8). On the other hand, recent findings suggest a potential benefit of calcium and vitamin D supplementation in cancer prognosis, particularly in reducing colorectal and breast cancer mortality. Similarly, post-diagnosis intake of specific antioxidants (such as vitamins C, D, and E) has shown correlations with decreased mortality among cancer survivors. However, these associations, primarily derived from observational studies, warrant cautious interpretation. Further research, especially randomized controlled trials, is essential to clarify these relationships, focusing on optimal dosages and the timing of supplementation post-diagnosis. Developing evidence-based recommendations for cancer survivors necessitates a comprehensive understanding of dietary supplement use alongside conventional treatments (9). The rationale for our deliberate choice of vitamins A, C, E, and D for inclusion in our comprehensive review is rooted in their remarkable attributes that make them ideal candidates for exploration in the context of therapeutic applications. First and foremost, these vitamins are readily available primarily through dietary consumption. This inherent accessibility renders them mainly practical for potential therapeutic interventions. Vitamin A can be found in numerous common foods such as carrots, sweet potatoes, and spinach. Vitamin C is abundantly present in fruits like oranges, strawberries, and kiwi, while vitamin E is found in nuts, seeds, and vegetable oil. Lastly, vitamin D can be synthesized by the skin when exposed to sunlight, and it is also available in fortified dairy products and fatty fish (10–13). Previous reviews of clinical trials, animal models, and *in vitro* studies suggest that dietary vitamins and minerals may contribute to the prevention and treatment of a variety of cancers such as those affecting the breast, lungs, liver, cervix, stomach, brain (glioma), lymphatic system (lymphoma), and prostate (14–26). However, further well-controlled studies are needed for a better understanding of their anticancer potential. In this review, we are going to shed light on the association between pharmacological vitamin doses and cancer and discuss the mechanisms of action. Additionally, we will briefly address how some studies have shown a lower incidence of cancer in vitamin-rich diets, which has received considerable attention in recent years.

## 2 Methods

Vitamins A, C, E, and D’s cancer treatment and prevention effects were examined in a comprehensive literature review. This investigation examined many human and animal scientific studies. Many petri dish and animal experiments were examined. This study examined how vitamins may treat and prevent cancer by reading many research papers. We searched PubMed, Google Scholar, Scopus, Web of Science, and other scientifically correct databases for the information we needed. With search terms like “vitamins,” “cancer treatment,” “prevention,” “chemotherapy,” and “immunotherapy,” we hoped to find relevant studies and learn from existing research. Researchers looked for English papers on how vitamins A, C, E, and D can treat or prevent cancer. Some studies examined how these vitamins might affect immunotherapy or chemotherapy. After reviewing all literature, 300 studies were found. After that, a strict process selected the best and most relevant studies. After a thorough review, studies that were not directly related to the topic, were not written in English, and did not provide enough experimental details or results were discarded. The best studies to examine and consider were 153. We were able to thoroughly examine the topic because we carefully selected and organized key information.

## 3 Biological activities of vitamins A, C, D, E and their impact on cancer prevention

Vitamins reflect a concept that involved different groups of bioactive compounds obtaining nutritional benefits for human health (27). Hence, they are one of the main essential nutrients that play a role in enhancing the immune system, improving mental health and the aging process, increasing energy levels, and protecting the cardiovascular system (28). Their biochemical functions in the body are translated by being coenzymes, hormones, antioxidants, promotors of signaling pathways, and modulators of cell and tissue growth and differentiation (29).

Interestingly, vitamin A (VA) showed an indirect impact on regulatory T cell development and cytokines production (30). Due to the chemical structure of VA and carotenoids (the presence of polyene chain), they have exhibited antioxidant activity by protecting cells from the damage of free radicals and singlet oxygen (31, 32). It was reported that dietary VA can improve glutathione peroxidase and superoxide dismutase activities as well as reduce reactive oxygen species (ROS) production (33). Retinol and its derivatives, found in animal-based foods, have essential roles in cell differentiation, proliferation, and apoptosis. They also play a key role in skin and bone growth through retinoic acid. Cytoplasmic binding proteins like CRBP-1 regulate intracellular retinoid levels, affecting retinol uptake and availability. Recent research has linked reduced CRBP-1 levels to increased malignancy in breast, ovarian, and nasopharyngeal cancers. Restoring CRBP-1 could enhance retinol sensitivity and reduce ovarian cancer cell viability, potentially guiding personalized retinoid therapy for cancer patients (34). Moreover, retinoids, when bound to RAR/RXR receptors (RAR: Retinoic Acid Receptor and RXR: Retinoid X Receptor), trigger a series of chromatin structure modifications that can encourage cell differentiation and initiate lasting epigenetic alterations. In the context of cancer cells, these changes hold the potential to induce differentiation toward a less malignant state. Retinoids achieve this by promoting stem cell differentiation and reshaping the gene expression patterns within tumor cells, rendering them more responsive to other therapeutic approaches. Consequently, retinoids are expected to play a significant role in future cancer treatments (35, 36).

On the other hand, Ascorbic acid is a powerful antioxidant in various reactions and metabolic processes. It neutralizes free radicals and toxins, subsequently attenuating oxidative damage and inflammatory responses (37, 38). Acting so, at micromolar concentrations, physiological ascorbate, a fully reduced form of vitamin C (VC), can reduce harmful ROS. Paradoxically, it also functions as a pro-oxidant at millimolar plasma concentrations via intravenous administration of pharmacological ascorbate (39). Besides, several studies have reported the potential of VC in reducing inflammation and modulating inflammatory biomarkers (40–43). A recent study has shown the complicated roles of VC in cancer biology, emphasizing its influence on stem cells, cancer metastasis, and immunotherapy. Vitamin C affects various biochemical reactions in cells, collagen synthesis, and the regulation of hypoxia-inducible factor (HIF), impacting extracellular matrix remodeling and cancer spread. It also displays promise in inhibiting cancer cell glycolysis and enhancing cancer immunotherapy when combined with anti-PD-L1 therapy (44). Beyond its antioxidant properties, VC directly impacts genomic stability, making it relevant in cancer prevention, stem cell therapy, and the treatment of hematological malignancies where TET mutations and aberrant DNA methylation are common (45). In recent years, the role of VC in cancer treatment has gained attention due to its influence on epigenetic regulation. Vitamin C, acting as an antioxidant and a co-factor for crucial enzymes like TET methylcytosine dioxygenases, plays a significant role in cancer therapy. Epigenetic abnormalities are common in cancer, and VC has consistently been shown to enhance DNA demethylation by TET enzymes when combined with DNA methyltransferase inhibitors (46).

As well, strong antioxidant properties are present in all vitamin E (VE) isomers (47). Because their chromanol ring contains phenolic hydrogen, they can scavenge reactive oxygen species (ROS). Vitamin E’s ability to scavenge reactive oxygen species (ROS) and prevent oxidative damage to cells and tissues is due to its phenolic hydrogen (48). Oxidative stress can cause free radical chain reactions that lead to lipid peroxidation in both people and animals used in experiments. Vitamin E is very important for stopping these free radical chain reactions, which stops lipid peroxidation and protects the integrity of biological membranes. As a strong antioxidant, vitamin E helps keep cells and tissues safe from the damage caused by oxidative stress (48). Many cancers, including skin and gastrointestinal cancers, may have oxidative stress as an underlying cause. A transcription factor known as Nrf2 regulates the induction of antioxidant enzymes. Research has demonstrated that specific forms of vitamin E, particularly γ-tocopherol and, to a lesser degree, α-tocopherol, can activate Nrf2. This, in turn, enhances the production of different antioxidant enzymes, such as SOD, catalase, glutathione peroxidase, and phase II detoxifying enzymes. In response to oxidative stress, cells launch an antioxidant response, which aids in cellular homeostasis maintenance (49). One reason why γ-tocopherol and δ-tocopherol are more effective than other tocopherol isoforms is because their chromanol rings have an unmethylated C-5 position. These isoforms are able to effectively combat reactive nitrogen species, such as NO2 and peroxynitrite, due to this structural feature. They can detoxify these reactive nitrogen species more effectively by producing 5-nitro-γ-tocopherol (50). Research has shown that vitamin E metabolites, like CEHCs (carboxyethyl hydroxychromans), can inhibit lipid peroxidation and have strong antioxidant capabilities. The free radical scavenging capabilities of these metabolites are even greater than those of the original vitamin E isoforms. Out of all the metabolites, γ-tocopheryl quinone is the one that triggers the antioxidant response the most effectively. This is accomplished by increasing glutathione levels and activating transcription factor 4 transcription. As a defense mechanism against oxidative stress, this antioxidant response shields cells from harm (51). Tocotrienols, like tocopherols, work as antioxidants by encouraging the production of different antioxidant enzymes, such as catalase and superoxide dismutase (SOD) (52). Tocopherols and tocotrienols are both very good at removing free radicals from membranes and lipoproteins. They can stop fatty acid peroxyl radicals from forming and turn them into tocopheroxyl radicals. These radicals are then broken down to make vitamin E again. This cycle of antioxidants helps keep membranes and lipoproteins intact and protects cell parts from oxidative damage (53, 54). It is widely recognized that eicosanoids, which are generated through the COX-2 and 5-LOX pathways, play a role in the advancement of colon cancer. Inhibiting these pathways is one mechanism by which vitamin E prevents carcinogenesis and reduces inflammation in the colon (55). It is well-established that eicosanoids produced by the COX-2 and 5-LOX pathways contribute to the progression of colon cancer. Therefore, vitamin E’s ability to inhibit these pathways contributes to its ability to inhibit colon inflammation and prevent carcinogenesis (55). A group of transcription factors known as peroxisome proliferator-activated receptors (PPARs) include PPAR-α, γ, and δ. These receptors control inflammatory pathways by blocking COX-2. In addition to PI3k/Akt and NF-κB, PPARs are linked to other signaling pathways. The ability of δ-tocopherol to activate PPARγ in various cell lines has been proven in studies conducted in both laboratory and living organism settings. Research has shown that δ-tocopherol inhibits inflammation, slows down cell cycle progression, and triggers cell death by activating PPARγ (56). In addition, research has shown that vitamin E (VE) can inhibit inflammatory markers through various pathways. Important inflammatory factors such as cyclooxygenase 2 (COX-2), tumor necrosis factor (TNF-α), and interleukin-6 (IL-6) are downregulated when prostaglandin E2 (PGE2) is inhibited (57).

Many studies have shown the correlation between vitamin D deficiency and the high incidence of infectious diseases and inflammatory autoimmune diseases (58, 59). Vitamin D reduces inflammation by mediating different pathways such as inhibition of NF-kB, increasing MKP5 expression, and blocking the prostaglandins pathway (60, 61). Regarding antioxidant activity, vitamin D was able to activate the Nrf2-Keap1 antioxidant pathway and improved nephropathy in a diabetic animal model (62). As well, VD supplementation can modulate oxidative stress parameters by reducing nitric oxide (NO), increasing glutathione (GSH) expression, and improving the total antioxidant capacity (63). Moreover, experimental findings indicate that the preventive effects of VD in cancer primarily result from its ability to influence critical biological processes, including cell proliferation, cell differentiation, the expression of growth factor genes, signal transduction, and apoptosis regulation (64). Additionally, VD has been found to interfere with the action of growth factors like insulin-like growth factors (IGF) by promoting the release of IGF binding protein 3 (IGFBP3), which limits the pro-proliferative effects of IGF (65). Vitamin D plays a crucial role in preventing early neoplastic processes through its anti-inflammatory, antioxidant, DNA repair, and cell regulation mechanisms. It also influences the intestinal microbiota, linked to inflammatory bowel diseases and colon cancer (66). It has shown promise in preventing and improving outcomes in colorectal cancer (CRC). Acting as a regulatory prohormone through its widespread vitamin D receptor (VDR) binding, it influences immune modulation and microbial composition in the colonic mucosa, affecting key mechanisms in CRC development (67). Calcitriol, known as bioactive vitamin D, plays a role in regulating numerous biological pathways. Its various actions, such as DNA damage repair and protecting against oxidative stress, can contribute to preservation of somatic stem cells. Additionally, calcitriol may inhibit the growth of cancer stem cells by inducing cell cycle arrest and promoting apoptosis (68). In summary, these vitamins collectively contribute to cancer defense and hold promise for improving cancer-related outcomes.

## 4 Vitamins as an anticancer agent: preclinical studies

### 4.1 Vitamin A

Vitamin A is a collection of life-essential, fat-soluble substances with an unsaturated isoprenoid main chain that is derived from both plants and animals. All derivatives of vitamin A share similar structural and physiological functions within the human body. Substances with a common structure of four isoprenoid subunits, whether of synthetic or natural origin, are also categorized as retinoids (69). Nevertheless, the core vitamin A sequence is concealed in their structures, and their activation of retinoid receptors is comparable to most of the other retinoids. Each of these substances is fat-soluble and, unlike water-soluble vitamins, accumulates rapidly in the body, particularly in the adipose tissue and liver. This is advantageous since temporary vitamin A deficiency is not linked with clinical symptoms, but on the other side, this can lead to accumulation as well as toxic effects (69). Vitamin A can be obtained from the diet either as provitamin A (carotenoids) through plants or as vitamin A (retinol and its near derivatives) through animal-based foods. The most common type of retinoid in the body is retinol. Furthermore, all-trans retinoic acid and 11-cis-retinal are the most active substances within the body (69).

Multiple mechanisms have been studied and proven for vitamin A, including more than gene signaling pathways in terms of anticancer effects (70), the effect of retinoids on mitochondrial functions (71), and other mechanisms and pathways that have been studied and proven (72, 73). All-trans retinoic acid, an endogenous retinoid, has been shown to inhibit the expression of PPARγ and to enhance the expression of DOK1 as it regulates the DOK1/PPARγ pathways thus inhibiting cell proliferation in breast cancer (MCF7 cell line) (74). As well, the synthetic retinoid (ST1926) had a greater effect on MDA-MB-231 and MCF7 cell lines represented by binding to β-catenin and cyclin D1, which regulates the Wnt/−catenin signaling pathway (75). Peretinoin, a synthetic retinoid, revealed a potential to inhibit hepatocarcinogenesis by downregulation of sphingosine kinase 1, which resulted in hindering cancer progression (76). It is also believed that the regulation of microRNAs is linked to retinoid anti-cancer mechanisms. An *in vitro* and *in vivo* study has shown the synergistic effect of all-trans-RA when combined with paclitaxel in A549 cell line. The difference in IC50 values between the combination and all-trans-RA alone was almost three times more efficient, which means that the cytotoxicity of the combination is higher. The researchers went further by testing this combination on solid tumors in mice, and it was proven that the treatment prolonged their survival rate and effectively prevented tumor progression (76). As well, all-trans-RA with a γ-secretase inhibitor (DAPT) as a combination therapy has shown better results than monotherapy in gastric cancer cells (MKN45 cells). Using MTT assay, a significant decrease in cell viability was reported. Additionally, it induced apoptosis by increasing caspases expression (77). An *in vitro* study demonstrated the antiproliferative activity of all-trans-RA-Podophyllotoxin conjugate against human gastric cancer cells (MKN-45 and BGC-823 cell lines). It inhibited cell growth by promoting apoptosis and blocking the ERK1/2 and AKT signaling pathways (78). In addition, all-trans-RA effectively reduced myeloid-derived suppressor cell (MDSC) accumulation and increased the frequency of CD8+ T cells in mice with cervical tumors. Notably, combining all-trans-RA with anti-PD-L1 antibody treatment resulted in further delayed tumor growth, along with an increased proportion of activated T cells and elevated levels of immune-stimulating cytokines in tumors (79). As well, all-trans-RA significantly inhibited hepatocellular carcinoma progression, reduced MDSCs and tumor-associated macrophages, and suppressed the expression of immunosuppressive molecules in the tumor microenvironment. In particular, it reduced the intratumoral infiltration G-MDSCs and the expression of protumor immunosuppressive molecules including arginase 1, iNOS, IDO and S100A8 + A9, which was accompanied by increased cytotoxic cell infiltration (80). Moreover, in a 4 T1 mouse model of breast cancer, researchers employed a combination of cryo-thermal therapy and all-trans-RA to address myeloid-derived suppressor cells (MDSCs). The combination significantly increased mouse long-term survival rates by promoting MDSC maturation, reducing suppressive molecule expression, and inhibiting metabolic pathways. Early after treatment, Th2 and Treg subsets decreased, improving Th1-dominant CD4+ T-cell differentiation, while later stages showed enhanced cytotoxicity of CD8+ T cells and natural killer cells. This approach offers promise for breast cancer therapy to overcome MDSC-induced immunosuppression (81). Chuang et al. suggested a synergistic therapeutic effect of polyinosinic-polycytidylic acid combined with 13-*cis*-retinoic acid in neuroblastoma (82). Using this combination in a mouse xenograft model led to suppressing tumor development through stimulating retinoic acid receptors beta (RAR-β), and preventing vessel formation (82).

### 4.2 Vitamin C

Vitamin C (VC) is a water-soluble natural compound, that has shown multiple mechanisms to target cancer. VC is being widely studied and this may be attributed to its high safety profile. It cannot be synthesized by the human body, and strictly and easily obtained from dietary intake to achieve a daily allowance of 75–90 mg per day. It can be obtained from fruits, like citrus fruits and berries, and vegetables like raw sweet pepper and broccoli, to achieve a plasma ascorbate concentration of 30–80 μM (12). Ascorbic acid is a powerful antioxidant in various reactions and metabolic processes. It neutralizes free radicals and toxins, subsequently attenuating oxidative damage and inflammatory responses (12, 83). VC has shown both preventive and anticancer capacities. As for its preventive capacity, studies have suggested a possible protective role of high VC intake with reduced colorectal cancer (84, 85), stomach cancer (86, 87), and pancreatic cancer (88). In addition, a recent *in vivo* study suggests that oral VC may have a preventive effect on the development of leukemia (89). Determining the adequate amount of oral VC is essential for optimizing human health and preventing chronic diseases including cancer.

Several studies have aimed to demonstrate the anticancer activity of VC alone or as adjunct therapies with conventional treatment methods. A study experimented VC in combination with each of eribulin mesylate, tamoxifen, fulvestrant, and trastuzumab on a variety of breast cancer cell lines (MCF7, SK-BR3, MDA-MB-231, and breast epithelial cells MCF10A), have shown that high-dose VC with each of those anti-cancer drugs had decreased the cell viability of cancer cells more comprehensively than high-dose of VC or chemotherapy alone (90). VC’s ability to reduce oxidative stress in order to manage the side effects of different cancer treatments has been explored in multiple studies (91, 92). An *in vitro* study has examined VC along with methotrexate (MTX) on their effectiveness against human glioblastoma multiforme (Human glioblastoma DBTRG cell line) (93). The results showed that VC alone (5 μM) is not cytotoxic to either DBTRG or HK-2 cells; however, in combination with 0.01 μM MTX was significantly able to reduce cell viability. Another *in vitro* study explored VC effects on oral squamous cell carcinoma (OSCC) using human OSCC-derived cell lines CAL27, SCC9, and SCC25 (94). Their finding suggested various mechanisms by which VC can induce anticancer effects. Initially, VC led to a significant rise in the ROS levels, inducing DNA damage and ATP depletion stresses. It also inhibited Bcl-2 expression and promoted Bax expression and caspase-3 cleavage. Additionally, VC induced cell cycle arrest at the G0/G1 phase in OSCC cells. Their data demonstrated a significant inhibitory impact of VC on the colony-forming ability of OSCC cells, dependent on concentration and time (94).

Another aspect of VC is its ability to modulate anticancer immune responses (39, 95). A study, using several mouse cancer models including colorectal, breast, melanoma, and pancreatic, explored the impact of daily intravenous high-dose VC (4 g/kg) (96). They observed that, in most cases, tumor growth was delayed only in the presence of a fully competent immune system, which suggests that VC antitumor activity is dependent on some immunomodulatory functions and not only on its pro-oxidant effects. Despite this being mostly an uncharted area, this study is evidence of VC’s capacity to modulate infiltration of the tumor microenvironment by cells of the immune system, delaying cancer growth in a T-cell-dependent manner. VC enhances the cytotoxic activity of adoptively transferred CD8 T cells, and also co-operates with immune checkpoint therapy (ICT) in many cancer types, proving that a combination of VC and ICT can be curative in models of mismatch repair deficient tumors with high mutational burden. To optimize the antiproliferative impacts of VC in murine tumors such as breast, colorectal, melanoma, and pancreatic cancers, a fully functional immune system is required (96).

Recent studies have provided a better mechanistic understanding of VC anti-cancer effects. VC has been proven to have anti-tumorigenic activity by increasing the reactive oxygen species in cancer cells without major toxicities. It can address three common vulnerabilities shared by many cancer cell, including redox imbalance, epigenetic programming, and oxygen-sensing regulation (39). Cancer cells tend to depict compensation mechanisms to survive. Antioxidants can be both destructive and accelerative of tumor progression. So, alone, it cannot prevent or suppress cancer. Hence the employment of pro-oxidant anticancer therapies, such as radiation, but serious collateral damage can occur causing a narrow therapeutic window. Ascorbate can overcome this issue via two common features of cancer cells: elevated levels of labile iron both extracellular and/or intracellular, and heightened dependence on glucose uptake and glycolysis. In the presence of redox-active transition metals, VC exhibits pro-oxidant effects, inducing oxidative stress through the formation of ROS or by inhibiting the antioxidant system (39, 97). In the process of cellular iron metabolism, the labile iron pool is chelatable and redox-active. When ferrous ion reacts with peroxide in those pools, they can produce the damaging hydroxyl radical •OH via the Fenton reaction. Ascorbate then comes to sustain this reaction by donating electrons to ferric ions generating redox active ferrous ions and perpetuating the generation of ROS in continuation that contributes to cell death. It was shown that Asc•− and peroxide were generated *in vivo* following intravenous ascorbate injections in rats (0.5 g/kg), and the production was ascorbate-dose-dependent (98). In an animal model study, daily high-dose ascorbate (4 g/kg) inhibited neuroblastoma growth, and tumors had increased activity of checkpoint kinase 2 (CHK2) and histone 2AX (H2AX). This implies that pharmacological ascorbate can induce DNA damage in tumors implanted in animals (99). Its selectivity toward cancer cells can be explained by different potential reasons. First, it has been shown in various types of cancer such as breast, prostate, and lymphoma that iron metabolism can be reprogrammed either by the upregulation of several iron intake pathways or the downregulation of iron export and storage pathways (100, 101). For instance, in breast cancer cells, the intracellular pool of labile ions is twice as elevated as in normal breast epithelial cells. Additionally, tumor cells may exhibit greater vulnerability to high-dose ascorbate compared to normal cells as they can generate higher levels of peroxide and •OH than their normal counterparts (39).

Oncogenic KRAS or BRAF mutations are contributors to the Warburg effect by upregulating GLUT1. This exploitation by cancer could serve as a potential therapeutic strategy for targeting the disease. When ascorbate is administered in high doses, it gets oxidized to DHA (Docosahexaenoic acid). DHA is structurally similar to glucose and thus, efficiently taken up via GLUT1 in KRAS or BRAF mutant cells. DHA is rapidly reduced back to ascorbate inside the cell. The decrease in intracellular antioxidants and subsequent rise in ROS levels interferes with glycolytic pathways, resulting in an energy crisis. For instance, recent studies demonstrated the selective elimination of gastric cancers and von Hippel–Lindau-null renal cancers, characterized by high GLUT1 expression, through high-dose ascorbate treatment (102, 103). Ascorbate has a significantly long half-life. It efficiently and continuously generates DHA in the tumors’ oxidative microenvironment. In addition, extracellular peroxide generated via the oxidation of ascorbate contributes to increased levels of DHA, as DHA is the main oxidized form of ascorbate. Normal erythrocytes are protected from high-dose ascorbate due to their increased level of antioxidant enzymes like GSH and have an enhanced glucose flux into the pentose phosphate (PP) pathway to generate more NADPH, which is a crucial molecule for the recovery of GSH. Patients who are deficient in G6PD, which is the rate-limiting enzyme in PP pathway, have a lagging in NADPH production, hindering those cells vulnerable to VC-induced oxidative stress, subsequently leading to erythrocytes death, thereby causing anemia (39).

Another way that VC acts to hinder cancer is its ability to activate 10-11 translocation (TET) proteins. Those proteins belong to the αKGDD enzyme family and they demethylate DNA and therefore, liberate the abnormal epigenetic reprogrammed methylated DNA seen in cancer. Ascorbate can donate an electron to a ferric ion and convert it to a ferrous ion, which is critical for TET activity. Ascorbate treatment *in vitro* increased TET activity, which led to DNA methylation, and subsequently liberated the expression of tumor suppressor genes, which increased chemosensitivity (104). An *in vivo* study has shown that daily injection of high-dose ascorbate (4 g/kg) in a leukemic mouse model, promoted DNA demethylation and the expression of genes critical for myeloid cell differentiation, and subsequently recapitulated the TET2 restoration phenotypes (105). Other αKGDDs such as JHDM and ALKB have yet an unclear role in cancer development and to what extent ascorbate influences their activity.

Usually, solid tumors obstruct and compress surrounding blood vessels, resulting in a hypoxic encounter. Tumor cells in their striving to survive, adapt to this hypoxic microenvironment. One of those oxygen-sensing regulations is the tumor cell’s activation of HIF1, an evolutionarily conserved transcription factor. HIF1 consists of two subunits, the O2-regulated HIF1α and a constitutively expressed HIF1β. Oxygen regulates HIF1α activity through “Proline hydroxylase domain proteins” (PHD) and HIF hydroxylases. Under normal oxygen concentration, HIF1 transcription is inhibited. Under hypoxic conditions, PHDs and HIF hydroxylases are inactive, leading to HIF1 activation. So, cells that lack adequate amounts of ascorbate can have increased HIF1α function, which may potentially play a role in tumor progression. HIF1 is an important target in cancer therapy via HIF hydroxylase that is enhanced by ascorbate treatment, thus suppressing tumor growth. Two studies support this hypothesis, indicating a mounting body of evidence that suggests inhibition of HIF1α-dependent tumor growth by ascorbate (106, 107). An *in vitro* study demonstrated that pharmacological ascorbate led to a reduction in the expression of HIF1α and its downstream target GLUT1 in thyroid cancer cells (108).

The previously mentioned findings have renewed people’s scope and vision of the pharmacological antitumor effects of VC, putting it in the spotlight for further elucidation of its function and mechanism.

### 4.3 Vitamin E

The compound known as α-tocopherol, often termed the “classic” form of vitamin E, has shown significant efficacy in traditional fertility restoration tests compared to other tocopherols. It is primarily present in wheat germ, almonds, and sunflower oil. On the other hand, γ-tocopherol is more commonly found in the American diet and is frequently present in vegetable oils like soybean, corn, and cottonseed (109). δ-Tocopherol is predominantly present in soybean and castor oils, with a comparatively lower concentration in wheat germ oil (52). The production of β-tocopherol (γ-TmT) frequently occurs as an incidental outcome in the course of vegetable oil refinement (110). Tocotrienols are commonly consumed in diets prevalent in East-South Asian regions, with palm and annatto oils serving as their primary sources (111).

A plethora of epidemiological research have investigated the association between the risk of cancer and vitamin E. Emerging studies indicate that vitamin E exhibits potential as a viable contender for cancer adjuvant therapy, primarily due to its anticarcinogenic characteristics. The initial hypothesis regarding the chemopreventive effects of vitamin E emerged due to the observation that individuals residing in the Mediterranean region, whose diets are rich in various isoforms of vitamin E, demonstrate a decreased susceptibility to colon cancer in comparison to individuals residing in Northern Europe and the United States (49, 112). Vitamin E exhibits an inhibitory effect on cell proliferation through the induction of apoptosis and cell cycle arrest. Apoptosis occurs via two distinct pathways: the extrinsic pathway, which is mediated by death receptor signaling, and the intrinsic pathway, which is impacted by mitochondrial rupture leading to the release of cytochrome c into the cytosol. In the end, these routes converge to trigger the activation of execution caspases, particularly caspase-3, resulting in the fragmentation of poly-ADP-ribose-polymerase (PARP) (52). Angiogenesis plays a crucial role in both the growth and spread of tumors, involving the rapid proliferation and migration of endothelial cells. Studies conducted in both laboratory settings (*in vitro*) and living organisms (*in vivo*) have provided evidence that certain isoforms of vitamin E, particularly tocotrienols, possess the capability to hinder the angiogenesis process (113). Additionally, it has been found that tocotrienols have the ability to decrease the expression of VEGF receptors. In a study conducted by Shibata et al., it was observed that tocotrienol administration led to the suppression of hypoxia-inducible factor (HIF-1), a transcription factor known to induce the expression of vascular endothelial growth factor (VEGF) and initiate angiogenesis in the presence of low oxygen levels. The study focused on human colorectal adenocarcinoma cells in an *in vitro* setting. The suppression of HIF-1 resulted in the blocking of the release of angiogenic factors (114). The proangiogenic cytokines interleukin-6 and interleukin-8 are well-known to elevate VEFG levels. Tocotrienol therapy resulted in a reduction of IL-6 and IL-8 levels in human umbilical vein endothelial cells (HUVEC cells) (115). Tocotrienols have demonstrated inhibitory effects on the phosphorylation of the PI3k/Akt signaling pathway, resulting in the downregulation of key signaling molecules including endothelial nitric oxide synthase (eNOS), glycogen synthase kinase 3 (GSK3), and extracellular signal-regulated kinase (ERK). This pathway plays a role in angiogenesis by promoting the binding interaction between vascular endothelial growth factor (VEGF) and its corresponding receptor (116).

In a comprehensive study investigating the anticancer effects of vitamin E variants, including natural (αT) and synthetic (γT) forms, on breast cancer models, γT emerged as particularly promising. *In vitro* experiments demonstrated γT’s efficacy in inhibiting colony formation and inducing apoptosis in various breast cancer cell types. The underlying mechanisms involved activation of caspases-8 and -9, coupled with downregulation of c-FLIP and survivin. Concurrently, assessments in a BALB/c mouse mammary cancer model revealed that both all-rac-αT and all-rac-αTAc significantly reduced tumor development and lung metastasis, highlighting their potential as anti-tumor and anti-metastatic agents in breast cancer. The study also compared αT, all-rac-αT, and all-rac-αTAc, with αT and all-rac-αT exhibiting lesser efficacy. The findings emphasize γT’s persistent and robust anticancer effects across *in vitro* and *in vivo* models, positioning it as a noteworthy candidate for breast cancer treatment. Further research is needed to unravel the molecular mechanisms and assess efficacy in clinical settings (117).

Research on β-tocotrienol (β-T3) showed its ability to lower PD-L1, a key immune checkpoint ligand, in both *in vitro* and *in vivo* settings. This decrease was caused by JAK2/STAT3 pathway inactivation. Results showed improved immune response and reduced PD-L1-induced tumor-intrinsic signaling, highlighting β-T3’s anticancer potential (118).

δ-Tocotrienol (δ-T3) has been widely recognized for its remarkable anticancer properties (119). In accordance with prior investigations, it has been observed that the administration of the antioxidant δ-tocotrienol (δ-T3) elicits apoptotic, paraptotic, and autophagic responses in castration-resistant prostate cancer (CRPC) cells (120). Recent findings show that δ-T3, a vitamin E variation, increases ROS and mitochondrial Ca2+ levels in PC3 and DU145 cell lines. This study aims to understand the mechanisms behind δ-T3’s anticancer effects on PC3 cells, focusing on the role of Ca2+ and ROS. The study examines how δ-T3 affects biological processes such as cell viability, apoptosis, paraptosis, autophagy, and mitophagy (121). Consistent with our earlier hypothesis, an elevated concentration of Ca2+ ions was identified as a contributor to the pro-death effects triggered by δ-T3. Interestingly, this excess Ca2+ did not influence autophagy and mitophagy processes in autophagy-defective DU145 cells. Additionally, our study reveals that δ-T3 retains its anticancer properties in this cell line, and notably, these properties persist despite an excessive accumulation of reactive oxygen species (ROS). This observation highlights the limited vulnerability of these cells to oxidative stress induced by ROS (121). Collectively, these discoveries demonstrate that δ-T3 induces structural and functional abnormalities in the mitochondria of CRPC cells, leading to cell death. These insights provide novel mechanistic understanding into the anticancer effects of this compound (121).

In a previous investigation, the utilization of mice as experimental subjects was undertaken to examine the levels of tocotrienol (T3) in plasma. T3, a member of the vitamin E family renowned for its anti-cancer attributes, was analyzed alongside a recently developed succinate ether derivative of T3 known as 6-O-carboxypropyl-α-tocotrienol (T3E). The primary objective of this study was to discern any potential disparities in pharmacokinetics between these two compounds (122). A mouse xenograft model was employed to evaluate the impact of oral administration of T3E on tumor growth in human malignant mesothelioma MM cells. Specifically, the H2052 cell line was utilized for this assessment (122). Tumor volume was significantly decreased in mice given 150 mg/kg T3E orally once every 2 days for 10 days without any reduction in body weight, indicating that T3E might have anti-MM effects (122). An *in vitro* study was undertaken to investigate the anticancer effects and mechanisms of action of methotrexate (MTX) in conjunction with vitamin E variants (tocopherol) and derivatives (tocopherol succinate) on triple-negative breast cancer (TNBC) (123). The study evaluated cell survival rates and protein levels using the MTT test and western blot analysis. Results indicated that combined therapy with MTX and tocopherol inhibited the growth of triple-negative breast cancer (TNBC) cells. Interestingly, varying doses of MTX exhibited distinct lethal effects on cells treated with tocopherol succinate. Higher MTX doses enhanced the anticancer activity of tocopherol succinate, while lower doses impeded it. Furthermore, the combined treatment demonstrated caspase-3 activation and poly (adenosine diphosphate ribose) polymerase cleavage in the treated cells (123). Against chondrosarcoma cells, a form of cartilage cell malignant primary bone tumor (124), Annatto tocotrienol (AnTT), -T3, and -T3 have anticancer properties. AnTT, -T3 and -T3 produced G1 arrest in SW1353 cells after 24 h of treatment, which escalated to apoptosis after 48 h. Furthermore, tocotrienol treatment caused considerable cytoplasmic vacuolation in SW1353 cells. The transcriptome analysis after tocotrienol therapy revealed enhanced signaling pathways in the endoplasmic reticulum stress, unfolded protein response, autophagy, and transcription (125).

### 4.4 Vitamin D

Vitamin D (VD) is a lipid-soluble vitamin that belongs to steroidal hormones (126). Vitamin D is found in two forms: vitamin D2 (ergocalciferol) and vitamin D3 (cholecalciferol) (127). Yeast and plants can produce ergocalciferol while humans obtain it from their diet. The human body can make vitamin D endogenously by exposing the skin to UV radiation from the sun and then converting cholesterol to vitamin D (128). Vitamin D can undergo different metabolism processes in the liver and kidney through the hydroxylation process. Vitamin D3 25-Hydroxyvitamin is produced by hydroxylation in the liver, whereas calcitriol (1,25-dihydroxycholecalciferol), the active form of vitamin D, is produced in the kidney (129). Calcitriol can bind to a special receptor called vitamin D receptor (VDR) and bind to DNA promoter sequences then control the gene expression. In addition, Calcitriol can play a major role in triggering Intracellular signaling such as Ca2+ and Cl− channels (130). In recent years, scientists conducted many studies and research to show either the effect of VD on cancer or enhancing the chemotherapy response. Vitamin D low levels significantly increased tumor growth in solid and non-solid types (131).

Most studies have found that the status of VD in serum can be a risk factor in various kinds of tumors for instance gastric, breast, colon, and prostate tumor (132). Furthermore, VD is important in controlling the tumorigenesis pathway, from admission to metastasis and microenvironment responses (64). Through a variety of mechanisms, including the regulation of cell behaviors like proliferation, differentiation, increased maturation and apoptosis, autophagy, and epithelial-mesenchymal transition (EMT), as well as the modulation of interactions between cells and their microenvironment, such as the suppression of inflammation, the immune system, angiogenesis, and anti-oxidants, vitamin D can control tumor cells (64). Furthermore, VD had been showed to activate apoptosis in cancer cells such as breast and carcinomas of the colon but not in a variety of prostate carcinoma cells, showing that various tumor cells react differently to vitamin D (133).

The animal model preclinical studies revealed that VD treatment is essential in controlling tumor development (134). A significant variable linked to tumor aggression in prostatic cancer (PCa) is the expression of Lysine-specific demethylase 1A (LSD1), which controls the expression of both estrogen and androgen receptors (ER) (135). Lysine-specific demethylase 1A (LSD1) regulates the transcriptional process of vitamin D receptors (VDR). They evaluated the role of LSD1 in VDR-mediated gene transcription for Cdkn1a, E2f1, Cyp24a1, and S100g using CWR22 cells transplanted in athymic nude mice via qRT-PCR-TaqMan. The cancer progression in xenograft mouse models correlated to the elevation of LSD1 and VDR protein levels (135). The findings showed that lowering LSD1 lowers PCa survival, and information on gene expression suggests that LSD1 has a dual co-regulatory function for VDR. LSD1 is regulating the 1, 25(OH)2D effects brought on by target genes by functioning as a coactivator and corepressor of VDR action. Translation (135).

In an *in vivo* examination, they used a non-immunodeficient MMTV-PyMT mice model for metastatic breast cancer to examine the effect of VD expression on breast tumor development. The study demonstrated that using exogenic of 25(OH)D slow down cancer growth with no hypercalcemic was observed (136). When compared to a normal diet (1,000 IU/kg), the non-immunodeficient MMTV-PyMT (mouse mammary tumor virus-polyoma middle tumor-antigen) mouse model of metastatic breast cancer with a low VD diet (25 IU/kg) demonstrated a significant advancement of mammary neoplasia (136). Moreover, systemic perfusion containing 1,25(OH)2D or 25(OH)D delayed the development of tumors and reduced lung metastasis. Equally metabolites decreased Ki-67, cyclin D1, and ErbB2 levels in cancer. In constant, the profusion of D 25(OH)obtained increasing levels of vitamin D up to 50% in tumor cells, which indicates effective activation and good cancer invasion. The obtained results in this study revealed that neoplasia, tumor growth, and metastasis can be delayed by vitamin D (136).

To determine if vitamin D-facilitated reduction of cell growth is associated with JNK1 (c-Jun NH2-terminal kinases), an *in vitro* study was carried out utilizing colorectal cells (137). The HT29 and Caco-2 human colon cancer cell lines as well as the HEK 293 T human embryonic kidney cell line were treated with calcitriol (1,25(OH)2-D3). Calcitriol was able to reduce cell creation in HT29 colon tumors and was linked with cell cycle stop. The phosphorylation of JNK1 and elevated expression of the cell cycle regulators p21 and p27 may be connected to the inhibitory effects of calcitriol (137). It has been shown unequivocally that JNK1 interacted with VDR both physically and functionally, gradually regulating both transcriptional and translational levels of VDR expression. The JNK1/VDR connection generated calcitriol-mediated suppression of cancer cell growth through JNK1 activation (137).

1,25-dihydroxyvitamin D (1,25(OH)2D) was discovered to predominantly modulate ROS and mitochondrial activity in an *in vitro* investigation employing osteosarcoma cells, which led to anticancer effects. The treatment led to reduced mitochondrial ROS and altered mitochondrial dynamics, while enhancing mitophagy and ROS defense mechanisms. Key molecular changes included the activation of tumor-suppressing pathways and the relocalization of the mTORC1 inhibitor DDIT4/REDD1, indicating a shift toward non-oxidative energy metabolism and reduce of tumor cell growth. This highlights the role of 1,25(OH)2D in controlling osteosarcoma cell creation through mitochondrial and redox balance regulation (138).

Moreover, it was found that effective vitamin D [(1,25(OH)2D3)] significantly hindered the growth of CD44-expressing human gastric cancer cells. Researchers linked the vitamin D impact to the Wnt/β-catenin signaling pathway and CD44 downregulation. Positive correlations were observed between CD44 and Vitamin D Receptor gene expressions in gastric tumor cells and patient tissues. In mouse model, both oral vitamin D intake and injections of 1,25(OH)2D3 overcame gastric cancer growing and CD44 expression, suggesting a potential therapeutic role for vitamin D in gastric cancer management and prevention (139).

Table 1 summarizes the preclinical studies of the mentioned vitamins.

**Table 1:** Preclinical studies of vitamins A, C, E, and D.

	Form of the vitamin	Type of cancer	Model of the study	Mechanism of action	References
Vitamin A	All-trans retinoic acid	Breast cancer	*In vitro* (MCF-7 cells)	↓expression of PPARγ	(74)
				↑stimulation of DOK1/PPARγ pathways	
				↓ cell proliferation	
	Synthetic retinoid	Breast cancer	*In vitro* (MCF-7 and MDA-MB-231)	↓ Wnt/−catenin signaling pathway	(75)
				↓ cell proliferation and differentiation	
	All-trans retinoic acid	Lung cancer	*In vitro* (Huh-7 cells)	↑ survival rate	(76)
			*In vivo* (SPHK1 knockout mice and SPHK1 transgenic mice)	↓ tumor progression	
	All-trans retinoic acid	Gastric cancer	*In vitro* (Human GC cell lines, AGS and MKN-45)	↓ cell viability	(77)
				↑ caspases	
				↑ apoptosis	
	All-trans-RA-Podophyllotoxin	Gastric cancer	*In vitro* (MKN-45 and BGC-823)	↓ cell growth	(78)
				↓ ERK1/2 and AKT signaling pathways	
				↑ apoptosis	
	Polyinosinic-polycytidylic acid/ 13-*cis*-retinoic acid	Neuroblastoma	*In vitro* [(SK-N-AS) (CRL-2137), SK-N-FI (CRL-2142), SK-N-DZ (CRL-2149), and BE(2)-M17 (CRL-2267)]	↑ stimulation of retinoic acid receptors beta (RAR-β)	(82)
			*In vivo* (nonobese diabetic/SCID) (NOD/SCID, NOD.CB17-Prkdcscid/NcrCrl) mice	↓ vessel formation	
	all-trans-RA	Cervical cancer	*In vivo* (BALB/c mice model)	↓ MDSC	(79)
				↑ CD + 8 T cells	
				↓ tumor growth	
	all-trans-RA	Hepatocellular carcinoma	*In vitro* (HepG2, Hepa1-6 and H22 hepatoma carcinoma cell line)	↓ MDSC	(80)
			*In vivo* (C57BL/6 N mice with Hepa1-6 tumor)	↓ tumor-associated macrophages	
				↓ arginase 1, iNOS, IDO, S100A8 + A9	
	all-trans-RA	Breast cancer	*In vivo* (4 T1 mouse model of breast cancer)	↑ mouse long-term survival rates	(81)
				↑ cytotoxicity of CD8+ T cells and natural killer cells	
Vitamin C		Breast cancer	*In vitro* (MCF10A, MDA-MB-231, MCF-7, and SK-BR-3)	↓ cell viability	(90)	
		Brain cancer	*In vitro* [human glioblastoma DBTRG and human renal tubular epithelial (HK-2)]	↓ cell viability	(93)	
		Oral cancer	*In vitro* (human OSCC-derived cell lines CAL27, SCC9, and SCC25)	↑ ROS level	(94)	
				↓ Bcl-2		
				↑ Bax expression, caspases		
				↑ cell cycle arrest at the G0/G1 phase		
		Colorectal cancer, breast cancer, melanoma, pancreatic cancer	*In vivo* (C57BL/6 J, BALB/c, FVB, and NOD-SCID mice)	↓ tumor growth	(96)	
				↑ CD8 T cells activity		
Ascorbate	Lymphoma	*In vitro* (DLBCL cell line OC-LY1)	↑ TET activity	(104)		
			↑ expression of tumor suppressor genes			
			↑ chemosensitivity			
Ascorbate	Leukemia	*In vivo* (Vav-tTA (VTA) transgenic mice)	↑ DNA demethylation	(105)		
			↑ TET2 restoration phenotypes			
Ascorbate	Thyroid cancer	*In vitro* (FTC133 and 8305c)	↓ expression of HIF1α and GLUT1	(108)
Vitamin E	β-tocotrienol	Prostate cancer and lung cancer	*In vitro* (Mouse Lewis lung cancer (LLC) cells, DU145 human prostate cancer cells and Jurkat T cells)	↓ PD-L1 expression	(118)
			*In vivo*	↓ JAK2/STAT3 pathway	
	δ-tocotrienol	Prostate cancer	*In vitro* (DU145 and PC3)	↑ ROS	(120)
				↑ apoptosis	
	Tocotrienol/6-*O*-carboxypropyl-α-tocotrienol	Malignant mesothelioma	*In vitro* (H2052 cell line) *In vivo* (ICR mice)	↓ tumor size	(122)
	Tocopherol/ tocopherol succinate	Breast cancer	*In vitro* (MDA-MB-231 and MDA-MB-468)	↓ cell growth	(123)
	Annatto tocotrienol/γ-tocotrinol/ δ-tocotrienol	Chondrosarcoma	*In vitro* (Human chondrosarcoma SW1353 cells)	↑ G1 arrest	(125)
				↑ apoptosis	
	(RRR-α-tocopherol [αT], RRR-γ-tocopherol [γT])	Breast cancer	*In vitro* (human MDA-MB-435, MCF-7, and murine 66 cL-4 mammary cancer cells)	↑ caspases-8 and -9	(117)
				↓ c-FLIP and surviving	
				↑ apoptosis	
				↓ colony formation	
	Synthetic forms (all-rac-α-tocopherol [all-rac-αT], all-rac-α-tocopheryl acetate [all-rac-αTAc])		*In vivo* (BALB/c mouse mammary cancer model)	↓ tumor growth	
				↓ metastasis	
Vitamin D	1,25(OH)_2_-D_3_	Prostate cancer	*In vitro* (C4-2 cells and CWR22 cells)	↑ tumor regression	(135)
			*in vivo* (athymic nude (Hsd:Athymic Nude-Foxn1nu) mice)	↓ LSD1	
	1,25-dihydroxycholecalciferol	Breast cancer	*In vitro*	↑ tumor regression	(136)
			*In vivo* (MMTV-polyoma middle T antigen (PyMT) transgenic mice)	↓ lung metastasis	
				↓ Ki-67, cyclin D1, and ErbB2 levels	
	Calcitriol	Colon cancer	*In vitro* (HT29 and Caco-2 cells)	↑ cell cycle arrest	(137)
				↑ expression of cell cycle regulators p21 and p27	
				↑ activation of JNK1	
	1,25-dihydroxyvitamin D (1,25(OH)2D)	Bone cancer	*In vitro* (Human MG-63 osteosarcoma cells)	↓ mTORC1	(138)
				↓ ROS	
	1,25(OH)2D3	Gastric cancer	*In vitro* (Human GC cells MKN45, MKN28, and KATO III)	↓ CD44	(139)
			*In vivo* (orthotopic GC nude mice model)	↓ Wnt/β-catenin signaling pathway	

## 5 Clinical trials

Numerous *in vitro* studies determined the importance of vitamins in preventing cancer initiation and empowering the immune system to defeat tumorigenesis. To support the preclinical results several clinical trials have been conducted using vitamins as adjuvant treatment with chemotherapy or immunotherapy.

### 5.1 Vitamin A

In a randomized, controlled, phase 3 trial, arsenic acid, and all-trans retinoic acid (45 mg/m^2^) treatment was applied in 235 patients with acute promyelocytic leukemia (140). The outcomes of this trial have shown that using this combination led to an increase in the cure rate and reduce relapse chances (140).

Another clinical trial demonstrated the effect of oral vitamin A (6,000 IU/day) in patients (n = 32) with low-risk gestational trophoblastic neoplasia who receive methotrexate chemotherapy (141). The intervention group exhibited a reduction of chemoresistance as well as a downregulation of the tumor marker β-hCG level (141). It was reported in a randomized double-blind study that using vitamin A supplementation (8,000 IU/8 h) with neoadjuvant chemotherapy (64 weeks) in 15 patients (total sample size is 30) has significantly improved the therapeutic response in the treatment of an advanced cervical carcinoma (142).

### 5.2 Vitamin C

Vitamin C is well-supported with studies that prove its direct potential to hinder cancer cell growth in preclinical models, yet there is limited clinical evidence regarding its anti-tumoral efficacy. Many studies have scrutinized the effects of pharmacological VC’s mechanisms on targeting vulnerable survival utilities of cancer cells that are essential for their perpetuating growth; however, the outcomes of high-dose intravenous VC in terms of its anti-cancer effects remain controversial. In the 1970s, clinical trials on patients with terminal cancer showed that intravenous high-dose ascorbate treatment, that achieved a peak plasma concentration of 6 mM, extended their survival (143, 144), whereas in the 1980s they failed to confirm these findings because ascorbate was given at the same dose orally and the peak plasma concentration was of less than 200 μM (145, 146). This was revealed with follow-up studies which concluded that the route of administration strongly affects VC pharmacokinetics, hence the discrepant results (147). A recent clinical study evaluated the addition of pharmacological ascorbate to platinum-based chemotherapy in patients with advanced-stage non-small cell lung cancer (NSCLC). This study was conducted on 38 patients who were administered 75 g ascorbate twice per week intravenously with carboplatin and paclitaxel every 3 weeks for four cycles. Reported results have shown that the addition of pharmacological ascorbate to platinum-based chemotherapy significantly improves tumor deterioration in the advanced stage of NSCLC, by meeting its primary endpoint with an objective response rate of 34.2% and reaching a disease-control rate of 84.2%. cytokine and chemokine levels propose that the studied combination induces an immune response by altering the host immune defenses (148). Another clinical study investigated the maximum tolerated dose (MTD) and recommended phase 2 dose (RP2D) of pharmacological ascorbic acid when it is combined with mFOLFOX6 or FOLFIRI with or without bevacizumab regimens in patients with metastatic colorectal cancer or gastric cancer. Thirty-six patients received 0.2–1.5 g/kg ascorbic acid infusion, in the dose-escalation phase, once daily (days 1–3) with mFOLFOX6 or FOLFIRI in a 14-day cycle until the MTD was reached. Followed by the speed-expansion phase in which pharmacological ascorbic acid was administered at the MTD, but the MTD was not reached and the RP2D was established to be 1.5 g/kg/day, days 1–3, with mFOLFOX6 or FOLFIRI. Results have reported 58.3% objective response rate, and 95.8% disease-control rate. This study has shown that combining pharmacological ascorbic acid with mFOLFOX6 or FOLFIRI has a significant decrease effect in all-grade and grade ≥ 3 bone marrow and gastrointestinal toxic effects (149).

### 5.3 Vitamin E

Furthermore, within the confines of a randomized controlled study, a total of 89 individuals who had been clinically diagnosed with differentiated thyroid cancer were subjected to I-131 radiation therapy. In the subsequent phase of the study, participants were assigned to receive various interventions, namely vitamin E (*n* = 30), vitamin C (*n* = 30), and supragingival scaling with vitamin C (*n* = 29). The present study aimed to investigate the potential protective effect of vitamin E on salivary glands, with a specific focus on enhancing parotid excretion function. The results obtained from the experimental analysis provide evidence supporting the notion that vitamin E indeed possesses a beneficial impact on salivary glands, particularly in terms of augmenting parotid excretion function (150). Vitamin E’s impact on lowering prostate cancer incidence has been demonstrated by two randomized controlled trials’ use of serum metabolomics analysis (151). Serum C22 lactone sulfate, a molecule strongly linked to the modulation of androgenic steroid metabolites, was found to be substantially reduced by a high-dose of vitamin E (400 IU/day) (151). On the other hand, the utilization of α-tocopherol and β-carotene as dietary supplements did not demonstrate a significant reduction in the likelihood of developing liver cancer or chronic liver disease (152).

### 5.4 Vitamin D

High serum levels of 25(OH)D were found to be associated with improved early-stage survival in non-small-cell lung cancer (NSCLC) in a randomized, double-blinded trial (153). The outcomes were classified into RFS and OS as primary and secondary, respectively. Referred to RFS was the period of time between the start date of the supplement and the earlier date of the cancer relapse or death from any cause. They referred to this period of time as OS, which is the start date of the supplement and the date of death from any cause. The overall results were no improvements in RFS and OS in the total study population, better OS in patients with high Vitamin D before taking the supplement (≥20 ng/mL) than the low level (<20 ng/mL) (153). When the study was limited to patients with early-stage adenocarcinoma and low 25(OH)D, the vitamin D group had significantly better 5-year RFS (86% vs. 50%, *p* = 0.04) and OS (91% vs. 48%, *p* = 0.02) than the placebo group. Among the polymorphisms studied, vitamin D binding protein (DBP1-rs7041) TT and CDX2 (rs11568820) AA/AG genetic constitution were associated with a well prediction, even after multivariable adjustment. Finally, vitamin D dietary supplementation may improve the prognosis of patients with lower 25(OH)D levels who have early-stage lung adenocarcinoma (153). Table 2 reviews the clinical studies of the mentioned vitamins.

**Table 2:** Clinical studies of vitamins A, C, E, and D.

Vitamins	Cancer type	Sample size	Study type	Type of vitamins and doses	The outcome of the study	References
Vitamin A	Acute promyelocytic leukemia	235	A randomized, controlled, open-label multicenter trial, phase 3 trial	All-trans retinoic acid (45 mg/m^2^)	Using all-trans retinoic acid with arsenic acid has increased the cure rate and reduced relapse chances	(140)
	Gestational trophoblastic neoplasia	32	A randomized, controlled clinical trial	oral vitamin A (6,000 IU/day)	The intervention group exhibited a reduction of chemoresistance as well as a downregulation of the tumor marker β-hCG level	(141)
	Advanced cervical carcinoma	30	A randomized double-blind study	Vitamin A supplementation (8,000 IU/8 h)	Improved therapeutic response of the chemotherapy	(142)
Vitamin C	Advanced-stage non-small cell lung cancer	38	Open-label, single-arm, non-randomized phase II study	Ascorbate (75 g twice per week) intravenously	Improved tumor deterioration and enhance immune response	(148)
	Metastatic colorectal cancer or gastric cancer	36	Phase 1 open-label, single-center, dose-escalation, and speed-expansion study	Ascorbic acid (0.2–1.5 g/kg) infusion	Improved therapeutic response of the chemotherapy and significantly decreased the side effects	(149)
Vitamin E	Thyroid carcinoma	89	A randomized, controlled clinical trial	NA	The results revealed the protective impact of vitamin E on salivary glands by enhancing parotid excretion function	(150)
	Prostate cancer	154	A randomized, placebo-controlled, double-blinded clinical trial	Vitamin E (400 IU/day)	Significantly decreased serum C_22_ lactone sulfate which is highly associated with modulating androgenic steroid metabolites	(151)
Vitamin D	non-small-cell lung cancer	155	A randomized, double-blind, placebo-controlled, parallel group trial	Vitamin D supplements (1,200 IU/day)	Patients with lower 25(OH)D levels who have early-stage lung adenocarcinoma may have a better chance of surviving if they take vitamin D supplements.	(153)

## 6 Critical assessment of vitamins safety and potential toxicity

In this section, we will assess the safety and potential toxicity of vitamins A, C, E, and D. By examining their toxicity profiles, we aim to provide a holistic perspective on their applications and shed light on the fine balance between their potential anticancer effects and the risk of toxicity when taken in excess.

Hypervitaminosis is a rapid-onset pathological condition caused by the excessive accumulation of any vitamin in the body. Specifically concerning vitamin A, hypervitaminosis is considered when the plasma concentration of retinol surpasses 2.09 μM. Toxicity is commonly associated with the improper use of dietary supplements but may also manifest following an elevated consumption of foods rich in preformed vitamin A, such as liver and eggs (154, 155). Hypervitaminosis A can occur in two forms: acute and chronic. Acute toxicity manifests within days to weeks after ingesting a few very high doses, typically more than 100 times the recommended dietary allowance (RDA). Symptoms include severe headache, blurred vision, nausea, dizziness, muscle aches, and coordination problems. Chronic hypervitaminosis A can result from regular consumption of high doses and may lead to dry skin, joint pain, fatigue, depression, and abnormal liver test results (10). Beta-carotene, a provitamin A, is generally regarded as safe when consumed through a balanced diet rich in fruits and vegetables. However, taking high-dose beta-carotene supplements may pose certain risks (156). Long-term, excessive beta-carotene intake, particularly in supplement form, has been associated with a higher risk of adverse health effects. Some studies suggest that high-dose beta-carotene supplements may be linked to an increased risk of lung cancer, particularly in individuals who smoke (157). Additionally, excessive beta-carotene intake can cause a harmless but noticeable condition called carotenemia, characterized by yellowish or orange discoloration of the skin (155).

Vitamin C has low toxicity, and excessive consumption is unlikely to cause serious adverse effects. The most commonly reported adverse effects include diarrhea, nausea, abdominal cramps, and other gastrointestinal disturbances due to the osmotic effect of unabsorbed vitamin C in the gastrointestinal tract. There is a theoretical concern that high vitamin C intake might lead to excess iron absorption, as vitamin C enhances nonheme iron absorption. Additionally, there is a possibility that vitamin C could act as a pro-oxidant, contributing to oxidative damage. Long-term intakes of vitamin C exceeding the tolerable upper intake level (UL) of 2,000 mg may increase the risk of adverse health effects (12). High doses of intravenous vitamin C or oral vitamin C can trigger hemolysis in individuals with glucose-6-phosphate deficiency. For those with paroxysmal nocturnal hemoglobinuria, oral ascorbic acid can exacerbate hemolysis. *In vitro* studies show that the impact of vitamin C on red blood cell lysis is concentration-dependent, worsening at low concentrations and inhibiting at high concentrations (158).

There is no evidence that high consumption of vitamin E in food can cause any adverse effects. However, it has been shown in animals that high doses of vitamin E supplements can cause hemorrhage and interrupt blood coagulation, and platelet aggregation inhibition. Consumption of 1,000 mg/day in adults appears to be safe, but yet, data are limited and based on small groups of people for short periods of time (11).

Excess consumption of vitamin D can be toxic, leading to marked hypercalcemia, hypercalciuria, and elevated serum 25(OH)D levels. Symptoms of vitamin D toxicity include nausea, vomiting, muscle weakness, neuropsychiatric disturbances, pain, loss of appetite, dehydration, polyuria, excessive thirst, and kidney stones. Toxicity is unlikely at daily intakes below 250 mcg, but even intakes below the UL may have adverse health effects over time. It is recommended to avoid serum 25(OH)D levels above 125–150 nmol/L (13).

## 7 Conclusion

In conclusion, the potential responsibility of vitamins A, C, E, and D in preventing and treating tumor is a subject of growing interest, supported by a combination of preclinical, clinical studies, and considerations of safety. Vitamins play complex roles in cancer prevention, influencing immune job, antioxidant resistance, inflammatory modulation, and epigenetic directive, which could enhance outcomes by affecting cell differentiation, proliferation, and apoptosis, and combating oxidative stress and DNA damage. Particularly, vitamin A with its regulatory role in cell growth and differentiation, impacts several signaling pathways linked to cancer. Vitamin C functions as an antioxidant, reducing oxidative stress and possibly enhancing immune responses against cancer cells. High-dose intravenous applications of vitamin C have shown potential in selectively targeting cancer cells. The antioxidative properties of vitamin E protect cell membranes from damage, with certain forms exhibiting anti-inflammatory effects. Vitamin D, through its role in cell differentiation and immune function, shows links to reduced risks in certain cancers like colorectal cancer. Interestingly, combining vitamins A, C, D, and E with chemotherapy and radiotherapy offers a customized strategy for cancer treatment. These vitamins enhance immune response, alleviate oxidative stress, inhibit angiogenesis, and induce apoptosis. The synergy between vitamins and conventional treatments provides promising paths for more effective and personalized therapies. Further research is essential to unlock their complete potential in cancer treatments.

Nevertheless, the current studies present limitations that must be acknowledged. Using different dosage regimens in human and animal studies may impact the mechanisms underlying the medicinal prospective of vitamins in tumors, often with complex, unpredictable outcomes. Although animal models offer preliminary insights, they do not always replicate human metabolic processes, potentially leading to different bioavailability and efficacy in humans. Additionally, *in vitro* findings, such as the observed toxicity of vitamins A and E to cancer cells, may not translate directly into therapeutic benefits *in vivo*, where complex biological systems can alter the vitamins’ impact on cancer cells.

The integration of preclinical and clinical research highlights the need for particular approaches based on cancer type, stage, and patient characteristics. However, there are significant gaps in our understanding, including determining optimal dosages for efficacy and safety, elucidating precise mechanisms of action, establishing clinical efficacy through more large-scale trials, developing personalized treatment approaches, and studying the longstanding effects of vitamin supplementation in cancer patients. Addressing these gaps through comprehensive, multidisciplinary research is crucial for fully realizing the potential of these vitamins in oncology.

## Author contributions

WT: Conceptualization, Project administration, Supervision, Validation, Visualization, Writing – review & editing. DA: Data curation, Software, Writing – original draft. ZA: Data curation, Methodology, Software, Writing – original draft. MJ: Data curation, Investigation, Software, Writing – original draft. LA: Conceptualization, Project administration, Writing – review & editing. RH: Data curation, Methodology, Software, Visualization, Writing – original draft. AM: Formal analysis, Investigation, Methodology, Software, Writing – original draft, Writing – review & editing.

## Figures and Tables

**Table:** 

DNA	Deoxyribonucleic acid
VA	Vitamin A
VC	Vitamin C
VE	Vitamin E
VD	Vitamin D
SOD	Superoxide dismutase
ROS	Reactive oxygen species
PCa	Prostatic cancer
LSD1	Lysine-specific demethylase 1A
VDR	Vitamin D receptors
AR	Androgen receptor
ER	Estrogen receptors
JNK1	c-Jun NH2-terminal kinases
HEK	Human embryonic kidney
DOK1	Docking protein 1
PPARγ	Peroxisome proliferator-activated receptor gamma
ERK1/2	The extracellular signal-regulated kinase 1/2
akt	Protein kinase B
RAR-beta	Retinoic acid receptor beta
HIF-1-alpha	Hypoxia-inducible factor 1-alpha
GLUT1	Glucose transporter 1,
PD-L1	Programmed death-ligand 1
JAK2	Janus kinase 2
STAT3	Signal transducer and activator of transcription 3
Ki-67	Antigen Kiel 67
MTD	Maximum tolerated dose
RP2D	Recommended phase 2 dose
OS	Overall survival
RFS	Recurrence-free survival
CRBP-1	Cellular Retinol-Binding Protein 1
RAR	Retinoic Acid Receptor
RXR	Retinoid X Receptor
IGF	Insulin-like growth factors
IGFBP3	Insulin-like growth factors binding protein 3
MDSCs	Myeloid-derived suppressor cell
RDA	Recommended dietary allowance
UL	Upper intake level
